# Obacunone protects retinal pigment epithelium cells from ultra-violet radiation-induced oxidative injury

**DOI:** 10.18632/aging.202437

**Published:** 2021-02-01

**Authors:** Da-Rui Huang, Chang-Ming Dai, Shu-Yan Li, Xiao-Feng Li

**Affiliations:** 1 Department of Ophthalmology, The Affiliated Huaian NO.1 People's Hospital of Nanjing Medical University, Huaian, China

**Keywords:** obacunone, UV radiation, retinal pigment epithelium cells, Nrf2 signaling, oxidative injury

## Abstract

Ultra-violet (UV) radiation (UVR) causes significant oxidative injury to retinal pigment epithelium (RPE) cells. Obacunone is a highly oxygenated triterpenoid limonoid compound with various pharmacological properties. Its potential effect in RPE cells has not been studied thus far. Here in ARPE-19 cells and primary murine RPE cells, obacunone potently inhibited UVR-induced reactive oxygen species accumulation, mitochondrial depolarization, lipid peroxidation and single strand DNA accumulation. UVR-induced RPE cell death and apoptosis were largely alleviated by obacunone. Obacunone activated Nrf2 signaling cascade in RPE cells, causing Keap1-Nrf2 disassociation, Nrf2 protein stabilization and nuclear translocation. It promoted transcription and expression of antioxidant responsive element-dependent genes. Nrf2 silencing or CRISPR/Cas9-induced Nrf2 knockout almost reversed obacunone-induced RPE cytoprotection against UVR. Forced activation of Nrf2 cascade, by Keap1 knockout, similarly protected RPE cells from UVR. Importantly, obacunone failed to offer further RPE cytoprotection against UVR in Keap1-knockout cells. *In vivo*, intravitreal injection of obacunone largely inhibited light-induced retinal damage. Collectively, obacunone protects RPE cells from UVR-induced oxidative injury through activation of Nrf2 signaling cascade.

## INTRODUCTION

Age-related macular degeneration (AMD) is one primary reason for vision loss and blindness among elderly people [1–3]. In the United States, AMD is detected in over eight million people [3]. For the vast majority of AMD patients with the “dry” or atrophic form, there is no effective therapy [1–3]. It is therefore urgent to explore novel and more efficient anti-AMD strategies.

In the pathology of AMD, Ultra-violet (UV) radiation (UVR) could induce substantial oxidative injury to human retina [1, 2, 4]. Sustained and intensive UVR will exert significant retinal damage, particularly in aged persons [5, 6]. Sunlight UV is divided into three segments based on the spectrum, including UVA, UVB, and UVC. Of which, UVC (below 286 nm) is filtered by the earth’s ozone layer. Most UVB (286-320 nm) is blocked by human cornea and lens (< 295 nm). UVA (320-400 nm) is the most eye-damaging UV spectrum, which transmits from human crystalline lens to retina [5, 6]. Melanin will efficiently absorb harmful UV in retina. However, melanin levels will drop significantly in aged people [5, 6]. Wearing sunglasses that block UV (<400 nm) could efficiently protect human retina from light damage [5]. Contact lenses can offer additional retinal protection by absorbing significant amounts of UVR [5].

Retinal pigment epithelium (RPE) is located between Bruch’s membrane and photoreceptors in human retina. It transports nutrients to photoreceptors from the choroid, and also transports wasters from the photoreceptors to the choroid [7–9]. Additionally, RPE cells are important for phagocytosis of photoreceptor outer membranes, maintaining blood-retina barrier, and polarized secretion of vascular endothelial growth factor (VEGF) [10, 11]. Among AMD patients, RPE atrophy, photoreceptors and Bruch's membrane dysfunction are commonly detected [7–9].

Studies have shown that short wavelength UVR, UVA2 (320-340 nm) and certain UVB (300-320 nm), induced significant oxidative injury in RPE cells. This will lead to impaired vision or even permanent blindness [5, 6, 12]. UVR induces profound reactive oxygen species (ROS) production and oxidative stress, causing lipid peroxidation and DNA damage, and eventually RPE cell death and apoptosis [13–19]. Conversely, antioxidants and ROS scavengers can efficiently protect RPE cells from UVR [14, 20–25].

We have previously shown that MIND4-17, a nuclear-factor-E2-related factor 2 (Nrf2) activator, protected RPE cells from UVR-induced oxidative stress [17]. Furthermore, PF-06409577 activated AMP-activated protein kinase (AMPK) signaling to inhibit UVR-induced RPE cell apoptosis [26].

Nrf2 cascade is a vital cellular endogenous antioxidant mechanism [27–30]. Without stimulation, Nrf2 binds Kelch-like ECH-associated protein 1 (Keap1), then ubiquitinated by a Cul3-based E3 ligase and degraded [27–30]. Activated Nrf2, as a transcription factor, will bind antioxidant responsive elements (ARE) to promote transcription and expression of antioxidant genes and phase II detoxification enzymes [27–30]. Nrf2-ARE dependent genes include *NAD(P)H quinone oxidoreductase 1 (NQO1)*, *heme oxygenase 1 (HO-1)* and several other important antioxidant genes [27–30]. Forced activation of Nrf2 signaling cascade, using genetic strategies or pharmacological agents, can efficiently protect RPE cells and other retinal cells from UVR and other oxidative insults [17, 26].

Obacunone is a highly oxygenated triterpenoid limonoid compound mainly found in the *Rutaceae* family plants (citrus and others). Obacunone has displayed multiple biological and pharmacological actions, including antioxidant [31], anti-inflammatory [32], anti-cancer [33], anti-obesity [34] and neuroprotective [35] activities. Its potential effect in RPE cells has not been studied thus far. The results of this study showed that obacunone activated Nrf2 cascade to protect RPE cells from UVR-induced oxidative injury.

## RESULTS

### Obacunone attenuates UVR-induced cytotoxicity in ARPE-19 cells and murine RPE cells

**Figure 1:**
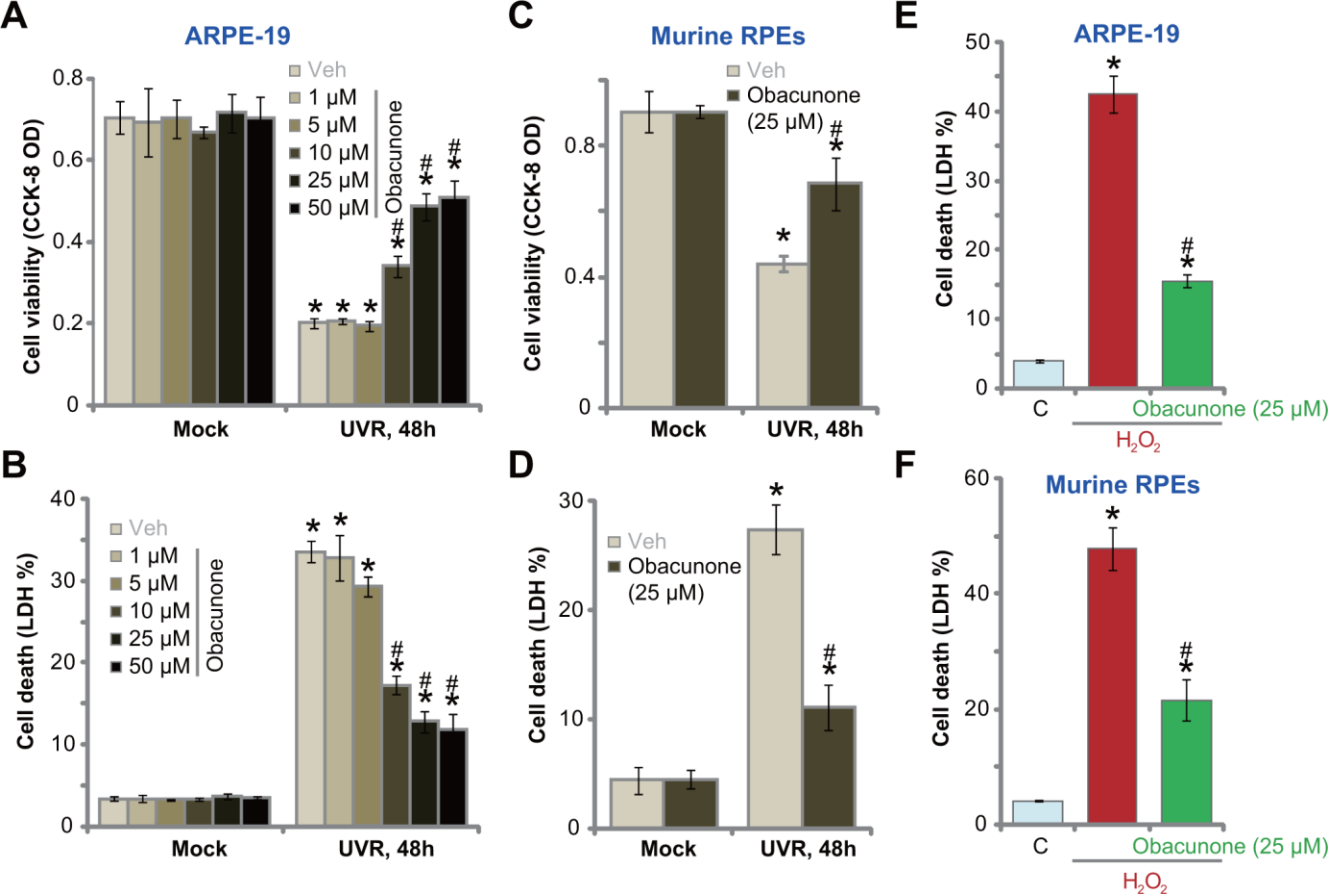
**Obacunone attenuates UVR-induced cytotoxicity in RPE cells.** ARPE-19 cells (**A**, **B**, **E**) or the primary murine RPE cells (**C**, **D**, **F**) were pretreated for 60 min with applied concentration of obacunone, cells were then subjected to UV radiation (UVR, UVA_2_+B, 30 mJ/cm^2^) or H_2_O_2_ (400 μM), and were further cultured for 48h; Cell viability was tested by CCK-8 assay (**A**, **C**), with cell death examined by LDH release in the conditional medium (**B**, **D**, **E**, **F**); Data were presented as mean ± SD (n=5). “Mock” stands for no UVR stimulation. “Veh” stands for the vehicle control. “C” stands for untreated control. *p < 0.05 *vs.* “Mock”/“C” treatment. ^#^ *p* < 0.05 *vs.* UVR/ H_2_O_2_ only treatment. Experiments were repeated three times, with similar results obtained.

**Figure 2:**
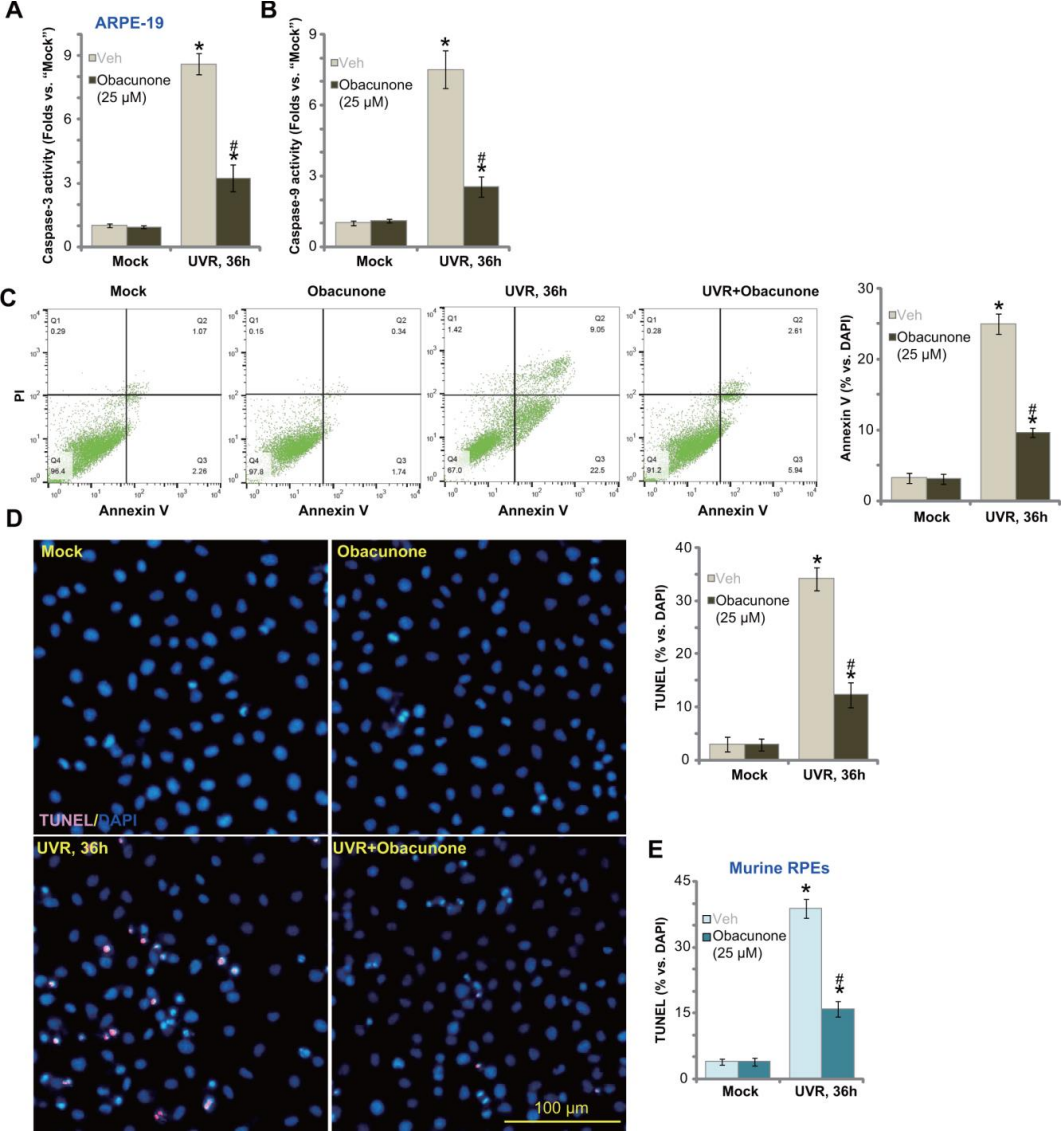
**Obacunone attenuates UVR-induced apoptosis in RPE cells.** APRE-19 cells (**A**–**D**) or primary murine RPE cells (**E**) were pretreated for 60 min with obacunone (25 μM), cells were then subjected to UV radiation (UVR, UVA_2_+B, 30 mJ/cm^2^), and further cultured for 36h, relative caspase-3/-9 activity (**A**, **B**) and apoptosis activation (**C**–**E**) were tested by the listed assays. Data were presented as mean ± SD (n=5). “Mock” stands for no UVR stimulation. “Veh” stands for the vehicle control. *p < 0.05 *vs.* “Mock” treatment. ^#^ *p* < 0.05 *vs.* “UVR” only treatment (with vehicle pretreatment). Experiments were repeated three times, with similar results obtained. Bar= 100 μm (**D**).

Obacunone has not been studied in retinal cells. The established ARPE-19 human RPE cells (from Dr. Jiang [36]) were treated with obacunone from 1-50 μM. The concentrations were determined based on the previous studies [37, 38] and our preliminary results. As demonstrated, obacunone treatment (1-50 μM) for 48h was safe for ARPE-19 cells, as it did not induce significant viability reduction (Figure 1A) and LDH release (Figure 1B). In line with the previous findings [18, 19, 36], UV radiation (UVR, 30 mJ/cm^2^) induced profound cytotoxicity in ARPE-19 cells, causing significant viability reduction (Figure 1A) and medium LDH release (Figure 1B). Significantly, pre-treatment with obacunone, at 10-50 μM, largely inhibited UVR-induced cytotoxicity in ARPE-19 cells (Figure 1A, 2B). Obacunone-induced RPE cytoprotection was dose-dependent, and ineffective at low concentrations (1-5 μM) (Figure 1A, 2B). Since 25 μM of obacunone effectively protected ARPE-19 cells from UVR (Figure 1A, 2B), this concentration was selected for further analyses.

In primary murine RPE cells, UVR led to potent viability reduction (Figure 1C) and cell death (Figure 1D), which were largely inhibited by obacunone (25 μM) as well (Figure 1C, 1D). Obacunone single treatment failed to induce death in murine RPE cells (Figure 1C, 1D). To mimic oxidative injury, hydrogen peroxide (H_2_O_2_) treatment (400 μM, for 48h) induced profound cell death in ARPE-19 cells (Figure 1E) and murine RPE cells (Figure 1F), which were largely attenuated with obacunone (25 μM) pretreatment (Figure 1E, 1F). These results showed that obacunone alleviated UVR-induced cytotoxicity in ARPE-19 cells and murine RPE cells.

### Obacunone attenuates UVR-induced apoptosis in ARPE-19 cells and primary murine RPE cells

UVR can induce apoptosis activation in ARPE-19 cells [18, 19]. Results in Figure 1 demonstrated that obacunone potently inhibited UVR-induced cytotoxicity in RPE cells, we next tested its effect on cell apoptosis. As shown UVR significantly increased caspase-3 (Figure 2A) and caspase-9 (Figure 2B) activity in ARPE-19 cells, which was largely inhibited by pre-treatment with obacunone (25 μM) (Figure 2A, 2B). Furthermore, significant apoptosis activation was detected in UVR-treated ARPE-19 cells, evidenced by increases in Annexin V-positive cell percentage (Figure 2C) and nuclear TUNEL staining (Figure 2D). In ARPE-19 cells, obacunone (25 μM) pretreatment potently inhibited UVR-induced apoptosis activation, decreasing ratios of Annexin V- and TUNEL-positive cells (Figure 2C, 2D).

TUNEL staining results, Figure 2E, confirmed that UVR-induced cell apoptosis in primary murine RPE cells was also largely attenuated by obacunone pretreatment. Obacunone single treatment failed to provoke cell apoptosis in ARPE-19 cells (Figure 2A–2D) and murine RPE cells (Figure 2E). These results showed that obacunone inhibited UVR-induced apoptosis in ARPE-19 cells and primary murine RPE cells.

### Obacunone ameliorates UVR-induced oxidative injury in ARPE-19 cells and primary murine RPE cells

**Figure 3:**
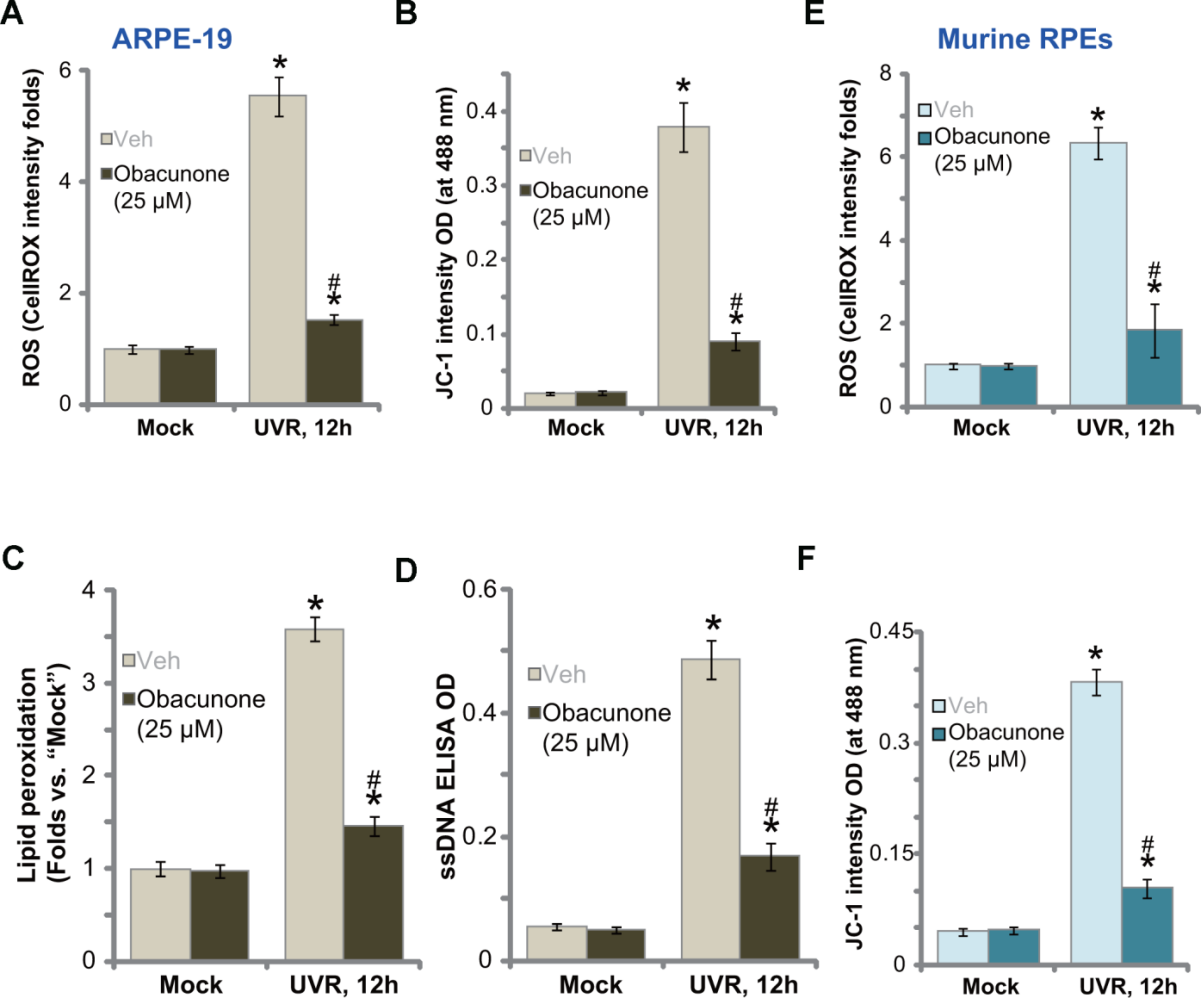
**Obacunone ameliorates UVR-induced oxidative injury in RPE cells.** ARPE-19 cells (**A**–**D**) or the primary murine RPE cells (**E** and **F**) were pretreated for 60 min with obacunone (25 μM), cells were then subjected to UV radiation (UVR, UVA_2_+B, 30 mJ/cm^2^), and were further cultured for 12h, ROS production (CellROX intensity, **A**, **E**), mitochondrial depolarization (JC-1 green monomers fluorescence intensity, **B** and **F**), lipid peroxidation (measuring relative TBAR activity, **C**) and DNA damage (measuring ssDNA contents, **D**) were tested, and results were quantified. Data were presented as mean ± SD (n=5). “Mock” stands for no UVR stimulation. “Veh” stands for the vehicle control. *p < 0.05 *vs.* “Mock” treatment. ^#^ *p* < 0.05 *vs.* “UVR” only treatment (with vehicle pretreatment). Experiments were repeated three times, with similar results obtained.

UVR induces significant ROS production and oxidative injury to RPE cells, a key mechanism mediating cell death [18, 19, 36]. Conversely, ROS scavengers or antioxidants can protect RPEs from UVR-induced cytotoxicity [18, 19, 36]. Obacunone could activate Nrf2 signaling cascade to fight against oxidative injury [38], we therefore tested whether obacunone can inhibit UVR-induced oxidative injury in RPE cells. Applying a CellROX dye assay [39, 40], we found that UVR induced significant ROS production in ARPE-19 cells (Figure 3A), which was accompanied by mitochondrial depolarization (JC-1 green monomers accumulation, Figure 3B). Obacunone pretreatment potently attenuated UVR-induced oxidative injury by suppressing CellROX intensity increase (Figure 3A) and mitochondrial depolarization (Figure 3B) in ARPE-19 cells. Further confirming oxidative injury, UVR induced significant lipid peroxidation (Figure 3C) and single strand DNA (ssDNA) accumulation (Figure 3D) in ARPE-19 cells, which were largely inhibited by obacunone as well (Figure 3C, 3D).

In the primary murine RPE cells UVR similarly induced ROS production (CellROX intensity increase, Figure 3E) and mitochondrial depolarization (JC-1 green monomers accumulation, Figure 3F). With obacunone pretreatment, UVR-induced oxidative injury was significantly attenuated (Figure 3E, 3F). Obacunone single treatment failed to induce oxidative injury in RPE cells (Figure 3A–3F). Therefore, obacunone potently inhibited UVR-induced oxidative injury in RPE cells.

### Obacunone activates Nrf2 signaling cascade in RPE cells

**Figure 4:**
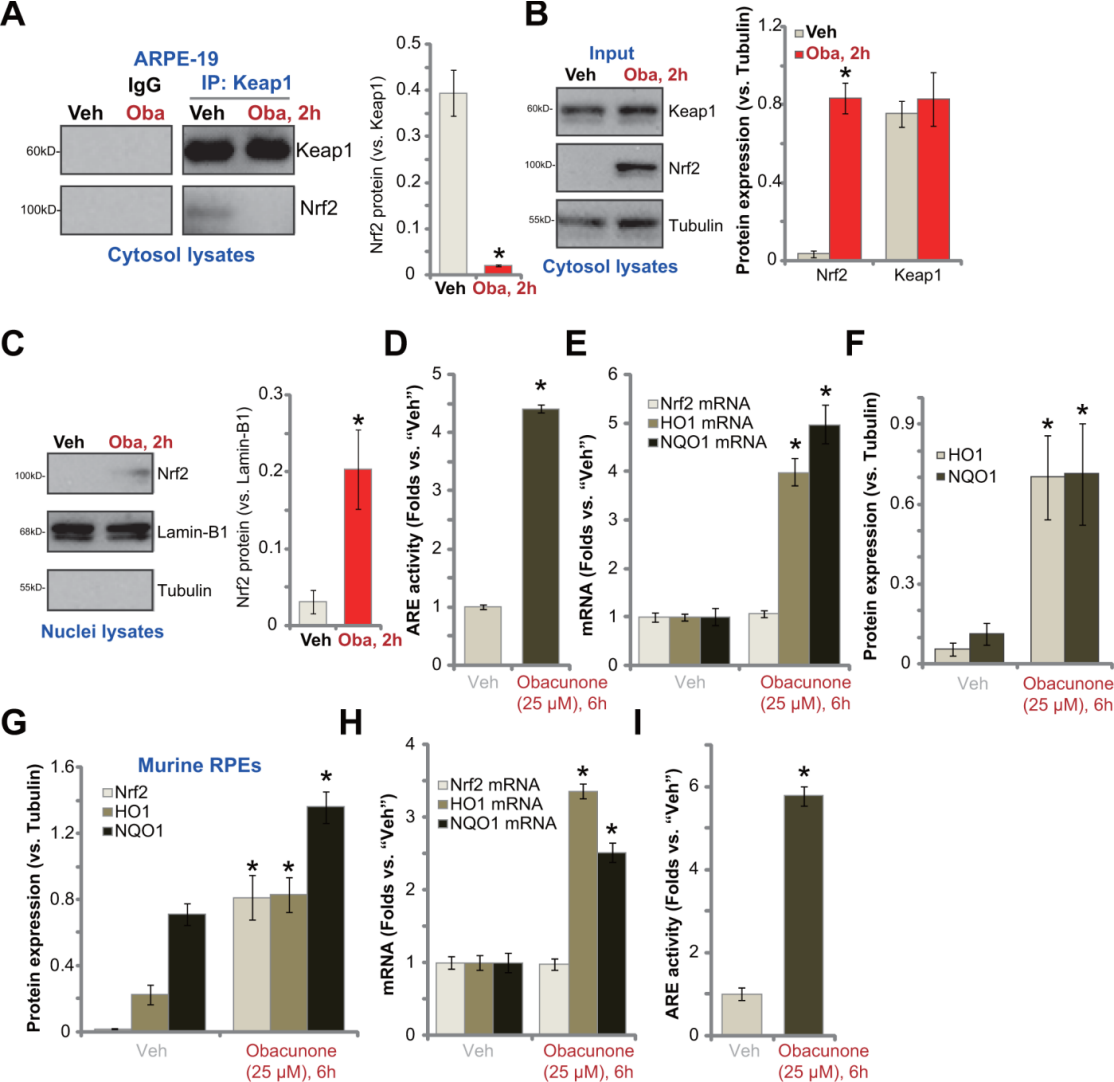
**Obacunone activates Nrf2 signaling cascade in RPE cells.** ARPE-19 cells (**A**–**F**) or the primary murine RPE cells (**G**–**I**) were treated with obacunone (“Oba”, 25 μM), cells were further cultured for applied time periods, the co-immunoprecipitation (Co-IP) assay was performed to test Keap1-Nrf2 association (**A**); Expression of listed proteins (in cytosol lysates and nuclear lysates) and mRNAs were tested by Western blotting (**B**, **C**, **F**, **G**) and qPCR (**E**, **H**) assays, and results were quantified; The relative ARE luciferase activity was tested as well (**D,** **I**). Expression of listed proteins was quantified, normalized to the loading control, and expressed as mean ± SD (n=5) (**A**–**C**, **F**, **G**). Data were presented as mean ± SD (n=5). “Veh” stands for the vehicle control. *p < 0.05 *vs.* “Veh” treatment. Experiments were repeated three times, with similar results obtained.

Forced activation of Nrf2 signaling can protect RPE cells from UVR-induced oxidative injury and cell apoptosis [17]. Since obacunone inhibited UVR-induced oxidative stress (Figure 3), we tested its potential effect on Nrf2 cascade in RPE cells. By applying a co-immunoprecipitation (Co-IP) assay, we showed that in ARPE-19 cells Keap1 immunoprecipitation with Nrf2 was disrupted with obacunone treatment (Figure 4A), resulting in Nrf2 protein stabilization and accumulation in cytosol fraction (Figure 4B). Keap1 protein levels were however unchanged (Figure 4B). Nrf2 protein also translocated to cell nuclei following obacunone treatment, evidenced by increased Nrf2 protein in the nuclear fraction lysates (Figure 4C). Importantly, obacunone treatment significantly increased ARE luciferase activity in ARPE-19 cells (Figure 4D) and promoted expression of ARE-dependent genes, including *HO1* and *NQO1* (Figure 4E). The protein expression of HO1 and NQO1 was upregulated as well following obacunone treatment (Quantified results in Figure 4F), with *Nrf2* mRNA expression unchanged (Figure 4E). Therefore, in ARPE-19 cells obacunone induced Keap1-Nrf2 disassociation, causing Nrf2 protein accumulation, nuclear translocation and expression of ARE-dependent genes.

In the primary murine RPE cells, obacunone treatment similarly induced Nrf2 protein accumulation (Figure 4G), expression of ARE-dependent genes (*HO1* and *NQO1*) (Figure 4G, 4H) and elevation of ARE luciferase activity (Figure 4I). It did not alter *Nrf2* mRNA expression (Figure 4H). Taken together, obacunone activated Nrf2 signaling cascade in RPE cells.

### Obacunone-induced RPE cytoprotection against UVR is abolished with Nrf2 silencing or knockout

**Figure 5:**
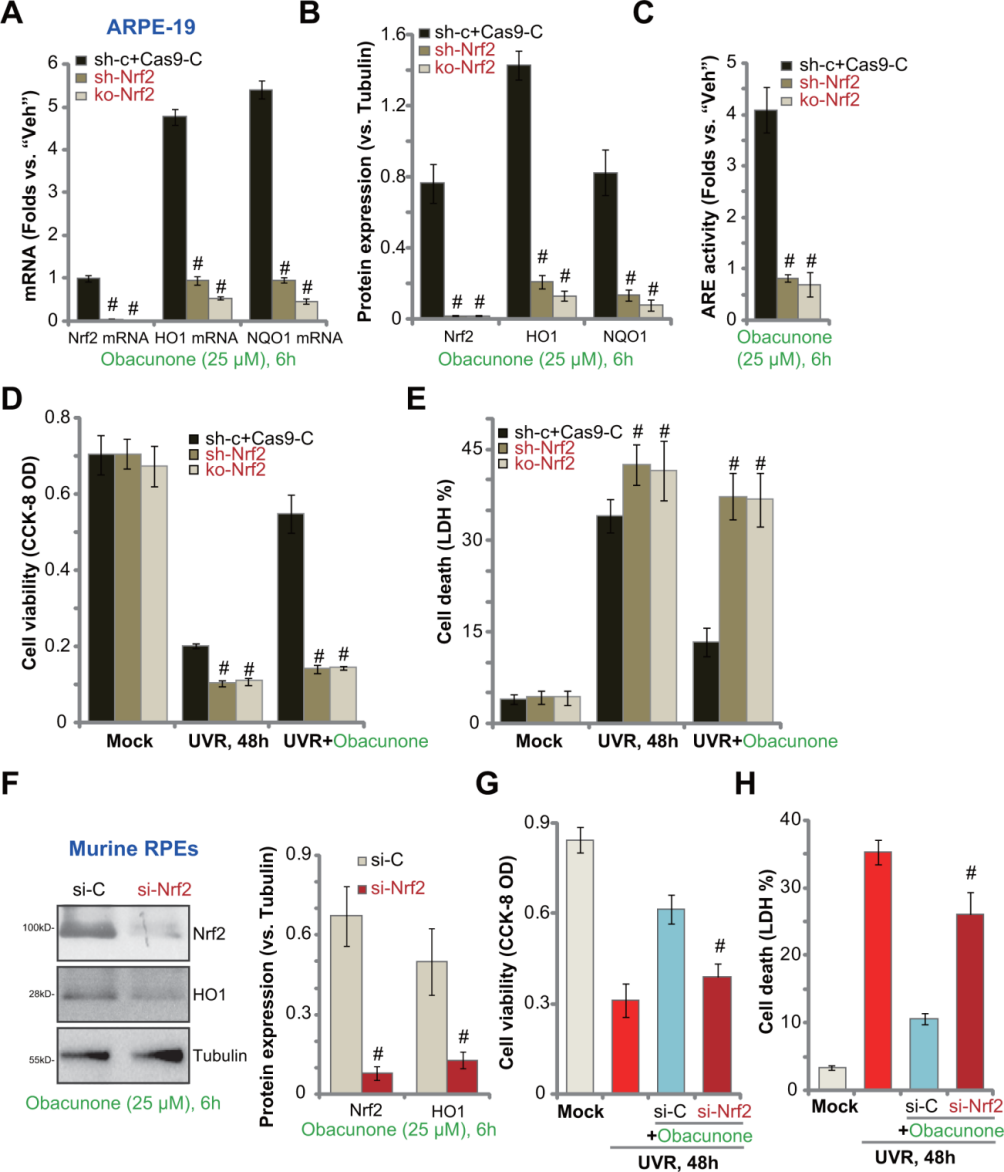
**Obacunone-induced RPE cytoprotection against UVR is abolished with Nrf2 silencing or knockout.** Stable ARPE-19 cells with Nrf2 shRNA (“sh-Nrf2”) or the CRISPR/Cas9-Nrf2-KO construct (“ko-Nrf2”), as well as the control cells (“sh-c+Cas9-C”), were treated with obacunone (25 μM), cells were further cultured for 6h, expression of listed genes was shown (**A**, **B**), with ARE activity tested (**C**). Alternatively, cells were pretreated with obacunone (25 μM) for 1h, followed by UVR and cultured for another 48h, cell viability and death were tested by CCK-8 (**D**) and medium LDH release (**E**) assays, respectively. The primary murine RPE cells were transfected with Nrf2 siRNA (“si-Nrf2”, 500 nM) or the scramble control siRNA (“si-C”, 500 nM), after 48h cells were treated with obacunone (25 μM) for another 6h, expression of listed proteins was shown (**F**). The murine RPE cells were pre-treated with obacunone (25 μM) for 1h, followed by UVR for another 48h, cell viability (**G**) and death (**H**) were tested. Expression of listed proteins was quantified, normalized to the loading control, and expressed as mean ± SD (n=5) (**B**, **F**). Data were presented as mean ± SD (n=5). ^#^ *p* < 0.05 *vs.* “sh-c+Cas9-C” cells (**B**–**E**). ^#^ *p* < 0.05 *vs.* “si-C” cells (**G**, **H**). Experiments were repeated three times, with similar results obtained.

If Nrf2 cascade activation is required for obacunone-induced RPE cell protection against UVR, Nrf2 depletion should reverse obacunone-induced actions. To test this hypothesis, ARPE-19 cells were transfected with Nrf2 shRNA lentivirus. Stable cells were established with selection by puromycin: sh-Nrf2 cells. Alternatively, a CRISPR/Cas9-Nrf2-KO construct was transduced to ARPE-19 cells. FACS sorting and puromycin selection were applied to establish stable cells: ko-Nrf2 cells. As compared to the control cells with scramble shRNA plus CRISPR/Cas9 control vector (“sh-c+Cas9-C”), *Nrf2* mRNA levels were dramatically decreased in sh-Nrf2 cells and ko-Nrf2 cells (Figure 5A). Consequently, obacunone-induced Nrf2 protein stabilization was abolished (protein expression results were quantified in Figure 5B). Furthermore, obacunone-induced mRNA (Figure 5A) and protein (Figure 5B) expression of HO1 and NQO1 was blocked by Nrf2 shRNA or KO. In sh-Nrf2 cells and ko-Nrf2 cells adding obacunone failed to increase ARE activity (Figure 5C).

As compared to the control cells, UVR-induced viability (CCK-8 OD) reduction (Figure 5D) and cell death (measuring medium LDH release, Figure 5E) were intensified in sh-Nrf2 and ko-Nrf2 ARPE-19 cells. Importantly, obacunone was ineffective against UVR in ARPE-19 cells with Nrf2 shRNA or KO (Figure 5D, 5E). Therefore, obacunone-induced ARPE-19 cytoprotection against UVR was reversed with Nrf2 shRNA or KO. These results suggested that activation of Nrf2 cascade is required for obacunone-induced actions.

In primary murine RPE cells, transfection of Nrf2 siRNA largely inhibited obacunone-induced Nrf2 protein stabilization (protein expression results were quantified in Figure 5F) and expression of HO1 (Figure 5F). With Nrf2 silencing, obacunone-induced RPE cytoprotection against UVR was compromised (Figure 5G, 5H). UVR-induced viability reduction (Figure 5G) and cell death (Figure 5H) were only slightly inhibited by obacunone pretreatment in Nrf2-silenced murine RPE cells.

### Keap1 depletion mimics obacunone-induced RPE cytoprotection against UVR

**Figure 6:**
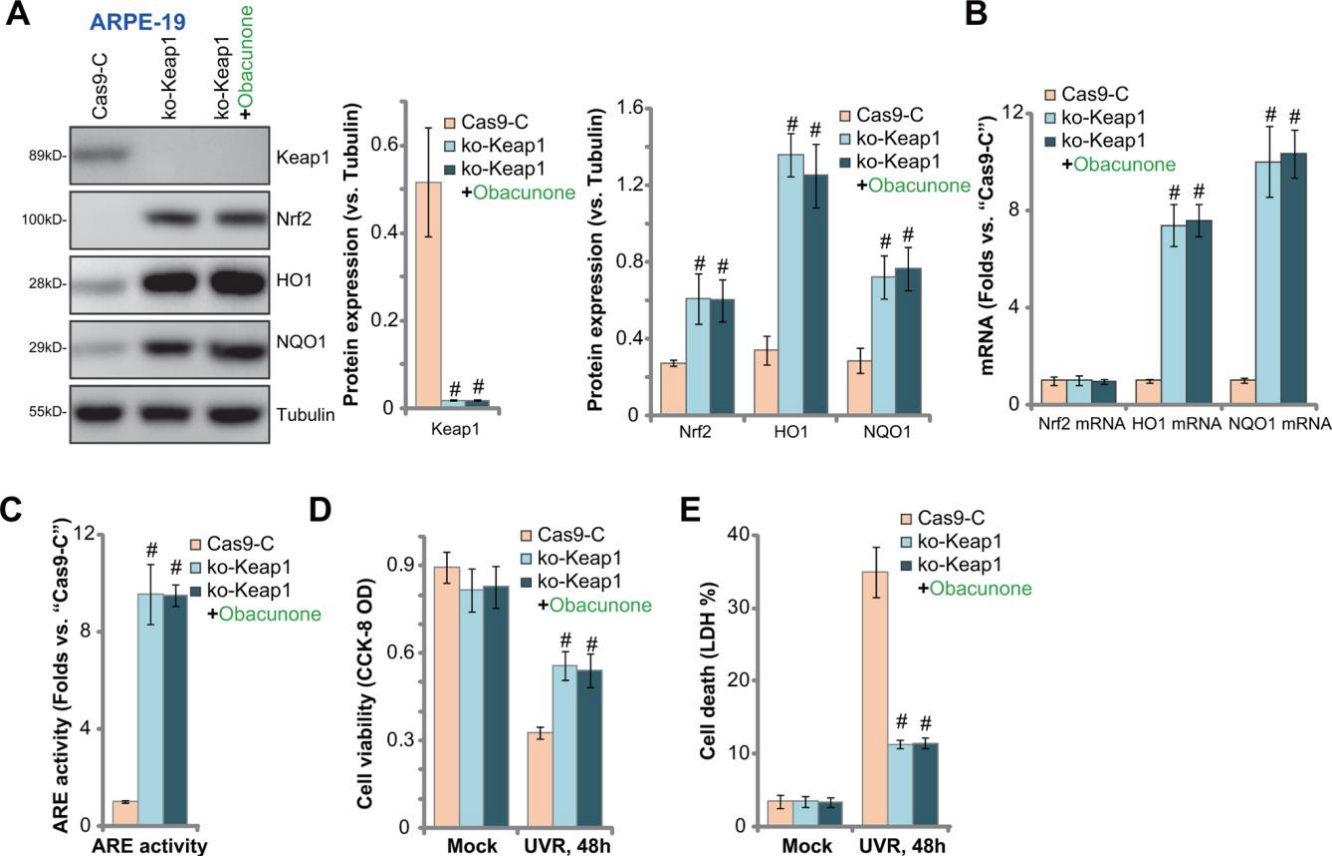
**Keap1 depletion mimics obacunone-induced RPE cytoprotection against UVR.** Stable ARPE-19 cells with the CRISPR/Cas9-Keap1-KO construct (“ko-Keap1”) or the empty vector (“Cas9-C”) were treated with or without obacunone (25 μM), cells were further cultured for 6h, expression of listed genes was shown (**A**, **B**), with ARE activity tested (**C**). Alternatively, cells were pretreated with obacunone (25 μM) for 1h, followed by UVR for another 48h, cell viability and death were tested by CCK-8 (**D**) and medium LDH release (**E**) assays, respectively. Expression of listed proteins was quantified, normalized to the loading control, and expressed as mean ± SD (n=5) (**A**). Data were presented as mean ± SD (n=5). ^#^ *p* < 0.05 *vs.* “Cas9-C” cells. Experiments were repeated three times, with similar results obtained.

Obacunone induced Keap1-Nrf2 disassociation and Nrf2 cascade activation, inhibiting UVR-induced oxidative injury and apoptosis in RPE cells. Therefore, Keap1 depletion should mimic obacunone-induced actions, activating Nrf2 signaling and protecting RPE cells from UVR. To test this hypothesis, a CRISPR/Cas9-Keap1 KO construct was transduced to ARPE-19 cells. Stable cells were established with FACS sorting and puromycin selection: “ko-Keap1” cells (Figure 6A). With Keap1 KO, Nrf2 protein was stabilized and accumulated (Figure 6A). HO1 and NQO1 mRNA and protein expression was significantly elevated (Figure 6A, 6B). *Nrf2* mRNA level was again unchanged (Figure 6B) but ARE activity increased (Figure 6C). Therefore, CRISPR/Cas9-induced Keap1 KO activated Nrf2 cascade in ARPE-19 cells.

The ko-Keap1 APRE-19 cells were protected from UVR, and presented with decreased viability reduction (Figure 6D) and cell death (Figure 6E), as compared to the control cells. Significantly, in ko-Keap1 RPE cells, obacunone treatment failed to further increase Nrf2 cascade activation (Figure 6A–6C), nor did it offer further cytoprotection against UVR (Figure 6D, 6E). Thus, obacunone was invalid against UVR in Keap1-KO cells with Nrf2 signaling pre-activated. These results further support that activation of Nrf2 cascade is required for obacunone-induced RPE cytoprotection against UVR.

### Obacunone protects mouse retina from light-induced damage

**Figure 7:**
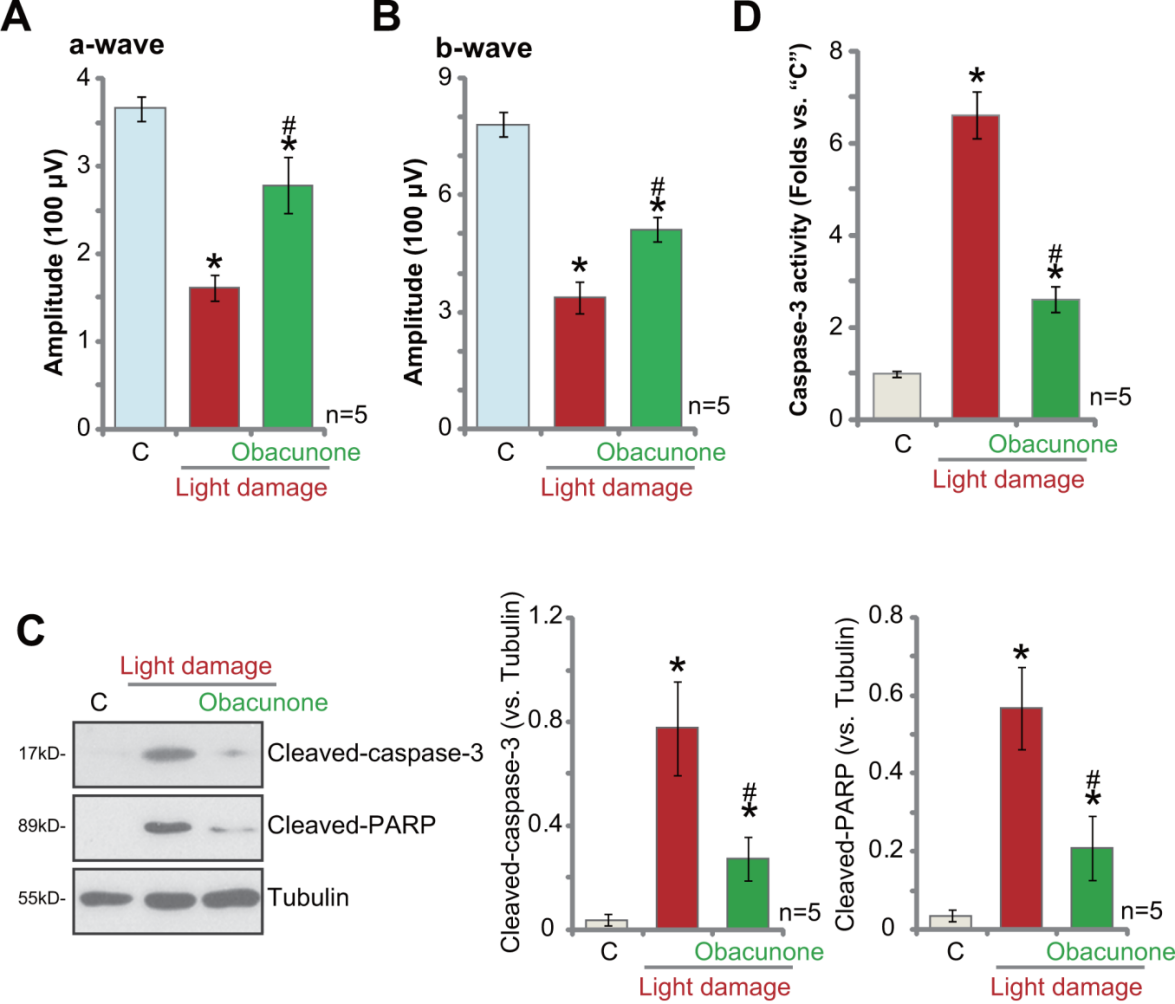
**Obacunone protects mouse retina from light-induced damage.** Twenty-four hours after the light exposure in mice retina, with or without obacunone intravitreal injection (2.5 mg/kg body weight, 1h pre-treatment), ERG was recorded, quantified amplitudes of a- and b-waves were presented (**A**, **B**). Retinal tissues were collected and expression of listed proteins was shown (**C**); The relative caspase-3 activity was tested as well (**D**). Data were expressed as mean ± standard deviation (SD, n=5). * *p* <0.05 *vs.* undamaged control mice (“C”). ^#^ *p* < 0.05 *vs.* “Light damage” only group (no obacunone administration).

At last, the potential effect of obacunone on mouse retina *in vivo* was studied. Using a previously-described light-induced retinal injury mouse model ([15, 21]), we showed that electroretinography (ERG) dysfunction reached peak 24h after the light exposure (Figure 7A, 7B) [41]. ERG b-wave amplitudes were tested from the trough of the a-wave to the peak of the b-wave, with the amplitudes of the a-wave measured from the initial baseline [15, 21, 41]. Significantly, intravitreal injection of obacunone (2.5 mg/kg body weight, 1h pre-treatment before light damage) potently alleviated light-induced reductions of both a- and b-wave amplitudes (Figure 7A, 7B). These results imply that obacunone protected mouse retina from light-induced damage.

Retinal tissues were collected and tested. As shown, 24h after light injury, levels of cleaved-caspase-3 and cleaved-poly (ADP-ribose) polymerase (PARP) (Figure 7C) as well as the caspase-3 activity (Figure 7D) were significantly increased in mouse retinal tissues. Importantly, intravitreal injection of obacunone potently inhibited apoptosis activation in the light-injured retinal tissues (Figure 7C, 7D).

## DISCUSSION

Studies have demonstrated that excessive and long-term UVR will induce significant oxidative injury and cytotoxicity in RPE cells [24, 25]. It is a major contributor of retinal degeneration in AMD pathology [1, 2, 4]. Conversely, antioxidants and ROS scavengers can protect RPE cells from UVR-induced cell injury [1, 2, 4]. Obacunone has displayed cytoprotective actions. For instance, Yoon et al., have shown that obacunone protected endothelial cells and restored endothelial function in ApoE-null mice [42]. Jeong et al., demonstrated that obacunone attenuated glutamate-induced oxidative injury in murine hippocampal neurons [35]. Furthermore, Zhou et al., reported obacunone inhibited high glucose-induced oxidative damage in renal tubular epithelial cells [38].

The results of the current study reported RPE cytoprotective activity of obacunone. Obacunone potently inhibited ROS accumulation, mitochondrial depolarization, lipid peroxidation and ssDNA accumulation in UVR-treated RPE cells. Furthermore, UVR-induced cell death and apoptosis were alleviated by obacunone. Therefore, obacunone exerted significant RPE cell protective activity by suppressing UVR-induced oxidative injury and apoptosis in RPE cells.

Our group and others have shown that activation of Nrf2 signaling can protect RPE cells from UVR [16, 17, 19, 36]. MIND4-17, a compound that activated Nrf2 signaling through blocking association with Keap1, protected RPE cells from UVR [17]. Recently, Chen et al., showed that by activation of Nrf2 signaling MIND4-17 protected retinal cells from high glucose-induced oxidative injury [36]. Tang et al., demonstrated that ginsenoside Rh3 activated Nrf2 signaling and protected RPE cells from UVR [19]. Ginsenoside Rh3 induced microRNA-141 expression, causing downregulation of Keap1 in RPE cells [19]. Interestingly, in RPE cells, forced overexpression of microRNA-141 activated Nrf2 signaling, inhibiting UVR-induced oxidative injury [16]. These results clearly indicated that forced activation of Nrf2 signaling cascade, using pharmacological or genetic methods, can protect RPE cells from UVR-induced oxidative injury.

Xu et al., have shown that obacunone can activate Nrf2 signaling in human cancer cells and immune cells, possibly by decreasing Nrf2 ubiquitination and increasing its stability [31]. In the present study we showed that obacunone activated Nrf2 signaling cascade in RPE cells, causing Keap1-Nrf2 disassociation, Nrf2 protein stabilization, cytosol accumulation and translocation to cell nuclei. Afterwards, it promoted transcription and expression of ARE-dependent genes (*NQO1* and *HO1*). Activation of Nrf2 cascade is required for obacunone-induced actions. As Nrf2 silencing or KO completely abolished obacunone-induced RPE cytoprotection against UVR. Furthermore, forced activation of Nrf2, through Keap1 KO, protected RPE cells from UVR-induced cell death. Importantly, obacunone was ineffective against UVR in Keap1-KO RPE cells. Together, obacunone inhibited UVR-induced oxidative injury in RPE cells through activation of Nrf2 signaling cascade.

Nrf2 can be used as a drug target for prevention and therapy of AMD and other retinal diseases related to oxidative stress. Agents that can inhibit or even reverse the development of geographic atrophy and prevent progression from atrophic to the “wet” form of AMD would be beneficial for patients. Here we found that intravitreal injection of obacunone largely inhibited light-induced retinal damage in mice. However, the current experimental results from cultured RPE cells and light-induced retinal injury mouse model could not be directly translated to human. Thus the efficacy and safety of obacunone against AMD and other retinal degeneration diseases will require further characterizations. Testing this compound with other administration routes, including oral administration or intravenous injection, in the mice model is certainly needed. Follow-up studies are required as well to test the pharmacokinetics and toxicology of this compound, using at the intravitreal route or other routes.

## MATERIALS AND METHODS

### Reagents and chemicals

Obacunone, hydrogen peroxide (H_2_O_2_), neomycin and puromycin were purchased from Sigma-Aldrich (St Louis, Mo). Antibodies for HO1 (#70081), NQO1 (#3187), Nrf2 (#12721), Keap1 (#8047), Tubulin (#2125) and Lamin B1 (#13435) were provided by Cell Signaling Tech (Shanghai, China). All cell culture reagents were from Gibco Co. (Suzhou, China).

### Cell culture

Cultures of the established human ARPE-19 cells were described early [17]. Primary murine RPE cells were provided by Dr. Jiang [21, 43], maintained under the previously-described protocol [21, 43]. This study is approved by the Institutional Animal Care and Use Committee (IACUC) and Ethics Committee of Nanjing Medical University.

### UV radiation

UV radiation (UVR, UVA2+B, 30 mJ/cm^2^) procedure was described early [26]. RPE cells were exposed to UVR for a total of 5 min. After the applied UVR procedure, RPE cells were maintained in basic medium for applied time periods.

### Cell viability and cell death assays

For testing cell viability, RPE cells were initially seeded into 96-well plates at 3,000 cells per well. After UVR, a Cell Counting Kit-8 (CCK-8, Dojindo Laboratories, Kumamoto, Japan) was utilized to test cell viability [17], with the CCK-8 optic density (OD) tested at 450 nm [44]. For cell death assays, RPE cells were initially seeded into six-well plates at 100,000 cells per well. Lactate dehydrogenase (LDH) release in the medium was tested by a commercial available kit (Takara, Tokyo, Japan), which was normalized to total LDH contents [17].

### Annexin V FACS

As described early [17], RPE cells were initially seeded into six-well plates at 150,000 cells per well. Following the applied treatment, cells were harvested, washed and resuspended in binding buffer with 1 μL of Annexin V-FITC and 1 μL of propidium iodide (PI) (Thermo-Fisher Invitrogen, Shanghai, China). After incubation for 15 min under the dark, cells were sorted by flow cytometry via CellQuest software (BD Biosciences, Shanghai, China). Annexin V ratio was recorded as a quantitative indicator of apoptosis intensity [43, 45].

### TUNEL assay

As described early [17], RPE cells were initially seeded into 12-well plates at 50,000 cells per well. After the applied treatments, RPE cells were stained with TUNEL and DAPI florescence dyes (Sigma). From ten random florescence images containing at least 1,000 cells per treatment, the TUNEL ratio (vs. DAPI staining) was calculated [46].

### ROS assay

RPE cells were initially seeded into 12-well plates at 50,000 cells per well. Following the applied treatments, RPE cells were incubated with 10 μM of CellROX for 30 min under the dark. The CellROX intensity was tested by a fluorescence spectrofluorometer (Molecular Devices, San Jose, CA).

### Caspase-3/-9 activity

RPE cells were initially seeded into 12-well plates at 50,000 cells per well. Following the applied treatments, 30 μg of cytosolic proteins per treatment were incubated with the caspase assay buffer [47] and the AFC-conjugated caspase-3/-9 substrate (Merck Millipore). AFC fluorescence was examined by a Fluoroskan Ascent FL machine.

### Single strand DNA (ssDNA) assay

RPE cells were initially seeded into six-well plates at 200,000 cells per well. Following the applied treatment, the ssDNA contents were tested by an ApoStrandTM ELISA apoptosis detection kit (BIOMOL International, Plymouth Meeting, PA).

### Mitochondrial depolarization

JC-1 fluorescence dye can aggregate to form green monomers in cells with mitochondrial depolarization [48]. RPE cells were initially seeded into 12-well plates at 50,000 cells per well. After the applied treatments, RPE cells were stained with JC-1 (10 μg/mL, Sigma) and its green intensity was examined at 488 nm using a fluorescence spectrofluorometer (Molecular Devices, San Jose, CA).

### Western blotting

The detailed protocols for the Western blotting assays were described previously [17, 49]. The same set of lysate samples were run in sister SDS-PAGE gels to test different proteins. For data quantification, the intensity (total gray) of each band was quantified using the ImageJ software (NIH), normalized to each loading control.

Co-Immunoprecipitation (Co-IP). Following the applied treatment a total of 800 μg protein lysates from APRE-19 cells were pre-cleared and an anti-Keap1 antibody was added. Keap1-bound proteins were captured with protein IgA/G beads and tested by Western blotting assays.

### Quantitative real-time PCR (qPCR)

The total cellular RNA was extracted using the TRIzol reagents (Thermo-Fisher Invitrogen), quantified and reversely transcribed [17]. Protocols for qPCR, using a SYBR Premix Ex TaqTM kit under the ABI-7700 fast PCR system (Takara Biotechnology, Japan), were described previously [17]. mRNA primers for human and murine *HO-1, NQO1*, *Nrf2* and *GAPDH* were described early [17, 50]. We employed a 2^-ΔΔCt^ method to calculate relative expression of targeted mRNA, *with GAPDH* as the reference gene and internal control.

### ARE reporter assay

RPE cells were initially seeded into six-well plates at 100,000 cells per well, transfected with the ARE-inducible firefly luciferase vector (from Dr. Jiang at Nanjing Medical University [51]). Following the applied treatment total cell lysates (30 μg per treatment) were subjected to ARE-reporter luciferase activity assay under a luminescence machine.

### Lipid peroxidation

RPE cells were initially seeded into six-well plates at 100,000 cells per well. Following the applied treatment, the thiobarbituric acid reactive substances (TBAR) activity, indicating lipid peroxidation, was examined using the previously-described protocol [17].

### Nrf2 shRNA

RPE cells were initially seeded into six-well plates at 150,000 cells per well. Nrf2 shRNA lentiviral particles (sc-37030-V, Santa Cruz Biotech, Shanghai, China) or the scramble control shRNA lentiviral particles (sc-108080, Santa Cruz Biotech) were added for 24h. Afterwards, puromycin (5.0 μg/mL) was added to select stable cells. Nrf2 knockdown was verified by Western blotting and qPCR assays.

### Nrf2 siRNA

The murine Nrf2 siRNA (sc-37049) and the scramble control siRNA were purchased from Santa Cruz Biotech. Murine RPE cells were cultured into six well tissue culture plates at 60% confluence, siRNA was utilized at 500 nM using Lipofectamine 2000 for transfection (last for 48h). Nrf2 silencing in primary murine RPE cells was verified by Western blotting.

### CRISPR-Cas9-mediated gene knockout

From Dr. Liu at Jiangsu University [52]. the lentiviral CRISPR-Cas9-Keap1-KO construct and the lentiviral CRISPR-Cas9-Nrf2-KO were obtained, each with GFP and puromycin selection gene. Each construct was transfected to cultured ARPE-19 cells (in complete medium with polybrene). Transfected ARPE-19 cells (GFP positive) were sorted by FACS, and monoclonal cells were further selected by puromycin to establish the stable cells. Keap1 KO or Nrf2 KO in the stable cells was verified by qPCR and Western blotting assays.

### Light damage to murine retinas

As reported [19] the BALB/c mice were provided by the Experimental Animal Center of CAS Shanghai (Shanghai, China). The detailed procedures of light exposure to mice retina were described early [15, 21]. Briefly, in each mice following the pupillary dilation retinas was exposed to 5000 lux of white fluorescent light [41]. One hour before light exposure obacunone (at 2.5 mg/kg body weight) was intravitreally injected to the right eye. For electroretinography (ERG) b-wave amplitudes were tested from the trough of the a-wave to the peak of the b-wave, with the amplitudes of the a-wave measured from the initial baseline [15, 21, 41]. The animal studies were performed according to the IACUC standards with ethical approval by Nanjing Medical University.

### Statistics

Results were presented as the mean ± standard deviation (SD). One-way ANOVA was employed to examine the significant differences between groups using SPSS 23.0 (SPSS, Chicago, CA). A two-tailed unpaired T test (Microsoft Excel 2007) was applied to test significance between two treatment groups. Values of *p* < 0.05 were considered statistically significant.

## References

[1] van Lookeren Campagne M, LeCouter J, Yaspan BL, Ye W. Mechanisms of age-related macular degeneration and therapeutic opportunities. J Pathol. 2014; 232:151–64. 10.1002/path.4266 24105633

[2] Beatty S, Koh H, Phil M, Henson D, Boulton M. The role of oxidative stress in the pathogenesis of age-related macular degeneration. Surv Ophthalmol. 2000; 45:115–34. 10.1016/s0039-6257(00)00140-5 11033038

[3] Jager RD, Mieler WF, Miller JW. Age-related macular degeneration. N Engl J Med. 2008; 358:2606–17. 10.1056/NEJMra0801537 18550876

[4] Young RW. Solar radiation and age-related macular degeneration. Surv Ophthalmol. 1988; 32:252–69. 10.1016/0039-6257(88)90174-9 3279560

[5] Roberts JE. Ultraviolet radiation as a risk factor for cataract and macular degeneration. Eye Contact Lens. 2011; 37:246–49. 10.1097/ICL.0b013e31821cbcc9 21617534

[6] Chalam KV, Khetpal V, Rusovici R, Balaiya S. A review: role of ultraviolet radiation in age-related macular degeneration. Eye Contact Lens. 2011; 37:225–32. 10.1097/ICL.0b013e31821fbd3e 21646979

[7] Fuhrmann S, Zou C, Levine EM. Retinal pigment epithelium development, plasticity, and tissue homeostasis. Exp Eye Res. 2014; 123:141–50. 10.1016/j.exer.2013.09.003 24060344 PMC4087157

[8] Chiba C. The retinal pigment epithelium: an important player of retinal disorders and regeneration. Exp Eye Res. 2014; 123:107–14. 10.1016/j.exer.2013.07.009 23880527

[9] Bhutto I, Lutty G. Understanding age-related macular degeneration (AMD): relationships between the photoreceptor/retinal pigment epithelium/Bruch’s membrane/choriocapillaris complex. Mol Aspects Med. 2012; 33:295–317. 10.1016/j.mam.2012.04.005 22542780 PMC3392421

[10] Nilsson SE, Sundelin SP, Wihlmark U, Brunk UT. Aging of cultured retinal pigment epithelial cells: oxidative reactions, lipofuscin formation and blue light damage. Doc Ophthalmol. 2003; 106:13–16. 10.1023/a:1022419606629 12675480

[11] Liang FQ, Godley BF. Oxidative stress-induced mitochondrial DNA damage in human retinal pigment epithelial cells: a possible mechanism for RPE aging and age-related macular degeneration. Exp Eye Res. 2003; 76:397–403. 10.1016/s0014-4835(03)00023-x 12634104

[12] Arunkumar R, Calvo CM, Conrady CD, Bernstein PS. What do we know about the macular pigment in AMD: the past, the present, and the future. Eye (Lond). 2018; 32:992–1004. 10.1038/s41433-018-0044-0 29576617 PMC5944649

[13] Roduit R, Schorderet DF. MAP kinase pathways in UV-induced apoptosis of retinal pigment epithelium ARPE19 cells. Apoptosis. 2008; 13:343–53. 10.1007/s10495-008-0179-8 18253836

[14] Yao J, Bi HE, Sheng Y, Cheng LB, Wendu RL, Wang CH, Cao GF, Jiang Q. Ultraviolet (UV) and hydrogen peroxide activate ceramide-ER stress-AMPK signaling axis to promote retinal pigment epithelium (RPE) cell apoptosis. Int J Mol Sci. 2013; 14:10355–68. 10.3390/ijms140510355 23685869 PMC3676843

[15] Gong YQ, Huang W, Li KR, Liu YY, Cao GF, Cao C, Jiang Q. SC79 protects retinal pigment epithelium cells from UV radiation via activating Akt-Nrf2 signaling. Oncotarget. 2016; 7:60123–32. 10.18632/oncotarget.11164 27517753 PMC5312373

[16] Cheng LB, Li KR, Yi N, Li XM, Wang F, Xue B, Pan YS, Yao J, Jiang Q, Wu ZF. miRNA-141 attenuates UV-induced oxidative stress via activating Keap1-Nrf2 signaling in human retinal pigment epithelium cells and retinal ganglion cells. Oncotarget. 2017; 8:13186–94. 10.18632/oncotarget.14489 28061435 PMC5355087

[17] Li C, Yan K, Wang W, Bai Q, Dai C, Li X, Huang D. MIND4-17 protects retinal pigment epithelium cells and retinal ganglion cells from UV. Oncotarget. 2017; 8:89793–801. 10.18632/oncotarget.21131 29163788 PMC5685709

[18] Xie L, Cheng L, Xu G, Zhang J, Ji X, Song E. The novel cyclophilin D inhibitor compound 19 protects retinal pigment epithelium cells and retinal ganglion cells from UV radiation. Biochem Biophys Res Commun. 2017; 487:807–12. 10.1016/j.bbrc.2017.04.128 28450114

[19] Tang CZ, Li KR, Yu Q, Jiang Q, Yao J, Cao C. Activation of Nrf2 by Ginsenoside Rh3 protects retinal pigment epithelium cells and retinal ganglion cells from UV. Free Radic Biol Med. 2018; 117:238–46. 10.1016/j.freeradbiomed.2018.02.001 29427790

[20] Cao G, Chen M, Song Q, Liu Y, Xie L, Han Y, Liu Z, Ji Y, Jiang Q. EGCG protects against UVB-induced apoptosis via oxidative stress and the JNK1/c-Jun pathway in ARPE19 cells. Mol Med Rep. 2012; 5:54–59. 10.3892/mmr.2011.582 21909619

[21] Li KR, Yang SQ, Gong YQ, Yang H, Li XM, Zhao YX, Yao J, Jiang Q, Cao C. 3H-1,2-dithiole-3-thione protects retinal pigment epithelium cells against Ultra-violet radiation via activation of Akt-mTORC1-dependent Nrf2-HO-1 signaling. Sci Rep. 2016; 6:25525. 10.1038/srep25525 27151674 PMC4858705

[22] Chen Y, Gibson SB. Is mitochondrial generation of reactive oxygen species a trigger for autophagy? Autophagy. 2008; 4:246–48. 10.4161/auto.5432 18094624

[23] Pacifici RE, Davies KJ. Protein, lipid and DNA repair systems in oxidative stress: the free-radical theory of aging revisited. Gerontology. 1991; 37:166–80. 10.1159/000213257 2055497

[24] Jin J, Ying H, Huang M, Du Q. Bioactive compounds in green tea leaves attenuate the injury of retinal ganglion RGC-5 cells induced by H2O2 and ultraviolet radiation. Pak J Pharm Sci. 2015; 28:2267–72. 26687755

[25] Guo D, Bi H, Liu B, Wu Q, Wang D, Cui Y. Reactive oxygen species-induced cytotoxic effects of zinc oxide nanoparticles in rat retinal ganglion cells. Toxicol In Vitro. 2013; 27:731–38. 10.1016/j.tiv.2012.12.001 23232460

[26] Li XF, Liu XM, Huang DR, Cao HJ, Wang JY. PF-06409577 activates AMPK signaling to protect retinal pigment epithelium cells from UV radiation. Biochem Biophys Res Commun. 2018; 501:293–99. 10.1016/j.bbrc.2018.05.003 29733844

[27] Krajka-Kuźniak V, Paluszczak J, Baer-Dubowska W. The Nrf2-ARE signaling pathway: An update on its regulation and possible role in cancer prevention and treatment. Pharmacol Rep. 2017; 69:393–402. 10.1016/j.pharep.2016.12.011 28267640

[28] Done AJ, Traustadóttir T. Nrf2 mediates redox adaptations to exercise. Redox Biol. 2016; 10:191–99. 10.1016/j.redox.2016.10.003 27770706 PMC5078682

[29] Zhang H, Davies KJ, Forman HJ. Oxidative stress response and Nrf2 signaling in aging. Free Radic Biol Med. 2015; 88:314–36. 10.1016/j.freeradbiomed.2015.05.036 26066302 PMC4628850

[30] Vriend J, Reiter RJ. The Keap1-Nrf2-antioxidant response element pathway: a review of its regulation by melatonin and the proteasome. Mol Cell Endocrinol. 2015; 401:213–20. 10.1016/j.mce.2014.12.013 25528518

[31] Xu S, Chen W, Xie Q, Xu Y. Obacunone activates the Nrf2-dependent antioxidant responses. Protein Cell. 2016; 7:684–88. 10.1007/s13238-016-0297-y 27530494 PMC5003787

[32] Gao Y, Hou R, Liu F, Liu H, Fei Q, Han Y, Cai R, Peng C, Qi Y. Obacunone causes sustained expression of MKP-1 thus inactivating p38 MAPK to suppress pro-inflammatory mediators through intracellular MIF. J Cell Biochem. 2018; 119:837–49. 10.1002/jcb.26248 28657665

[33] Chidambara Murthy KN, Jayaprakasha GK, Patil BS. Obacunone and obacunone glucoside inhibit human colon cancer (SW480) cells by the induction of apoptosis. Food Chem Toxicol. 2011; 49:1616–25. 10.1016/j.fct.2011.04.014 21515332

[34] Ono E, Inoue J, Hashidume T, Shimizu M, Sato R. Anti-obesity and anti-hyperglycemic effects of the dietary citrus limonoid nomilin in mice fed a high-fat diet. Biochem Biophys Res Commun. 2011; 410:677–81. 10.1016/j.bbrc.2011.06.055 21693102

[35] Jeong GS, Byun E, Li B, Lee DS, Kim YC, An RB. Neuroprotective effects of constituents of the root bark of Dictamnus dasycarpus in mouse hippocampal cells. Arch Pharm Res. 2010; 33:1269–75. 10.1007/s12272-010-0818-9 20803131

[36] Chen N, Li Y, Huang N, Yao J, Luo WF, Jiang Q. The Nrf2 activator MIND4-17 protects retinal ganglion cells from high glucose-induced oxidative injury. J Cell Physiol. 2020; 235:7204–13. 10.1002/jcp.29619 32020639

[37] Xie J, Zhang AH, Qiu S, Zhang TL, Li XN, Yan GL, Sun H, Liu L, Wang XJ. Identification of the perturbed metabolic pathways associating with prostate cancer cells and anticancer affects of obacunone. J Proteomics. 2019; 206:103447. 10.1016/j.jprot.2019.103447 31326558

[38] Zhou J, Wang T, Wang H, Jiang Y, Peng S. Obacunone attenuates high glucose-induced oxidative damage in NRK-52E cells by inhibiting the activity of GSK-3β. Biochem Biophys Res Commun. 2019; 513:226–33. 10.1016/j.bbrc.2019.03.201 30954216

[39] Sung HK, Song E, Jahng JW, Pantopoulos K, Sweeney G. Iron induces insulin resistance in cardiomyocytes via regulation of oxidative stress. Sci Rep. 2019; 9:4668. 10.1038/s41598-019-41111-6 30874600 PMC6420583

[40] Celeghini EC, Alves MB, de Arruda RP, de Rezende GM, Florez-Rodriguez SA, de Sá Filho MF. Efficiency of CellROX deep red^®^ and CellROX orange^®^ fluorescent probes in identifying reactive oxygen species in sperm samples from high and low fertility bulls. Anim Biotechnol. 2019. [Epub ahead of print]. 10.1080/10495398.2019.1654485 31424334

[41] Shibagaki K, Okamoto K, Katsuta O, Nakamura M. Beneficial protective effect of pramipexole on light-induced retinal damage in mice. Exp Eye Res. 2015; 139:64–72. 10.1016/j.exer.2015.07.007 26213307

[42] Yoon J, Park M, Lee Jh, Min BS, Ryoo S. Endothelial nitric oxide synthase activation through obacunone-dependent arginase inhibition restored impaired endothelial function in ApoE-null mice. Vascul Pharmacol. 2014; 60:102–09. 10.1016/j.vph.2014.01.006 24509132

[43] Zhang H, Liu YY, Jiang Q, Li KR, Zhao YX, Cao C, Yao J. Salvianolic acid a protects RPE cells against oxidative stress through activation of Nrf2/HO-1 signaling. Free Radic Biol Med. 2014; 69:219–28. 10.1016/j.freeradbiomed.2014.01.025 24486344

[44] Ren K, Lu X, Yao N, Chen Y, Yang A, Chen H, Zhang J, Wu S, Shi X, Wang C, Sun X. Focal adhesion kinase overexpression and its impact on human osteosarcoma. Oncotarget. 2015; 6:31085–103. 10.18632/oncotarget.5044 26393679 PMC4741590

[45] Yang L, Zheng LY, Tian Y, Zhang ZQ, Dong WL, Wang XF, Zhang XY, Cao C. C6 ceramide dramatically enhances docetaxel-induced growth inhibition and apoptosis in cultured breast cancer cells: a mechanism study. Exp Cell Res. 2015; 332:47–59. 10.1016/j.yexcr.2014.12.017 25576381

[46] Zhang G, Wang C, Sun M, Li J, Wang B, Jin C, Hua P, Song G, Zhang Y, Nguyen LL, Cui R, Liu R, Wang L, Zhang X. Cinobufagin inhibits tumor growth by inducing intrinsic apoptosis through AKT signaling pathway in human nonsmall cell lung cancer cells. Oncotarget. 2016; 7:28935–46. 10.18632/oncotarget.7898 26959116 PMC5045368

[47] Zhao LP, Ji C, Lu PH, Li C, Xu B, Gao H. Oxygen glucose deprivation (OGD)/re-oxygenation-induced in vitro neuronal cell death involves mitochondrial cyclophilin-D/P53 signaling axis. Neurochem Res. 2013; 38:705–13. 10.1007/s11064-013-0968-5 23322110

[48] Brooks MM, Neelam S, Fudala R, Gryczynski I, Cammarata PR. Lenticular mitoprotection. Part A: Monitoring mitochondrial depolarization with JC-1 and artifactual fluorescence by the glycogen synthase kinase-3β inhibitor, SB216763. Mol Vis. 2013; 19:1406–12. 23825920 PMC3695757

[49] Wang JY, Jin X, Zhang X, Li XF. CC-223 inhibits human head and neck squamous cell carcinoma cell growth. Biochem Biophys Res Commun. 2018; 496:1191–96. 10.1016/j.bbrc.2018.01.168 29402408

[50] Yu M, Li H, Liu Q, Liu F, Tang L, Li C, Yuan Y, Zhan Y, Xu W, Li W, Chen H, Ge C, Wang J, Yang X. Nuclear factor p65 interacts with Keap1 to repress the Nrf2-ARE pathway. Cell Signal. 2011; 23:883–92. 10.1016/j.cellsig.2011.01.014 21262351

[51] Chen ZJ, Rong L, Huang D, Jiang Q. Targeting cullin 3 by miR-601 activates Nrf2 signaling to protect retinal pigment epithelium cells from hydrogen peroxide. Biochem Biophys Res Commun. 2019; 515:679–87. 10.1016/j.bbrc.2019.05.171 31178131

[52] Liu H, Feng Y, Xu M, Yang J, Wang Z, Di G. Four-octyl itaconate activates Keap1-Nrf2 signaling to protect neuronal cells from hydrogen peroxide. Cell Commun Signal. 2018; 16:81. 10.1186/s12964-018-0294-2 30442144 PMC6238317

